# Effect of obesity on fragility fractures, BMD and vitamin D levels in postmenopausal women. Influence of type 2 diabetes mellitus

**DOI:** 10.1007/s00592-022-01923-x

**Published:** 2022-07-04

**Authors:** M. J. Gómez de Tejada-Romero, P. Saavedra-Santana, F. de la Rosa-Fernández, N. Suárez-Ramírez, A. Martín-Martínez, F. Martín del Rosario, M. Sosa-Henríquez

**Affiliations:** 1 Research Group On Osteoporosis and Mineral Metabolism, University of Las Palmas de Gran Canaria, Las Palmas de Gran Canaria, Spain; 2 Department of Medicine, University of Seville, Seville, Spain; 3 Department of Mathematics, University of Las Palmas de Gran Canaria, Las Palmas de Gran Canaria, Spain; 4 Gynecology and Obstetrics Service, Maternal-Infant Insular University Hospital Complex, Las Palmas de Gran Canaria, Spain; 5 Bone Metabolic Unit, Maternal-Infant Insular University Hospital Complex, Las Palmas de Gran Canaria, Spain

**Keywords:** Obesity, Diabetes Mellitus type 2, Vitamin D, Fragility fractures, Osteoporosis

## Abstract

**Aims:**

To see the effects of obesity on risk fracture, bone density (BMD), and vitamin D levels in a group of postmenopausal women, and consider how comorbid type 2 diabetes mellitus (T2DM) modifies them.

**Methods:**

679 postmenopausal women were grouped into obese and non-obese. Obese women were grouped into those with T2DM and those without. 25(OH)-vitamin D, PTH and BMD were measured, and prevalent fragility fractures were gathered.

**Results:**

Obese women had higher prevalence of T2DM, than non-obese women. Levels of 25(OH)-vitamin D were lower and those of PTH higher in obese women, BMD values were higher in obese women. Diabetic-obese women had a higher prevalence of non-vertebral fractures than non-diabetic-obese. Multivariate logistic regression model showed association of fragility fractures with age, total hip BMD, BMI and T2DM. Obese women have higher BMD and lower 25(OH)-vitamin D values (and higher PTH) than non-obese, without diabetes.

**Conclusions:**

T2DM confers an increased risk of non-vertebral fractures in postmenopausal obese women.

## Introduction

Obesity and type 2 diabetes mellitus (T2DM) are two important health problems with a high prevalence in the world population [1]. Multiple studies have also shown a relationship between both pathologies and bone metabolism: first, the well-demonstrated positive correlation between body mass index (BMI) and bone mineral density (BMD) [2]. This, together with the “padding” effect of the fat tissue to absorb the force of the impact in falls, made it was assumed that obese patients had a lower risk of suffering from osteoporotic fracture [3].

However, over the past decade some studies found that obesity had a positive association with fracture, particularly in postmenopausal women [4, 5]. Some studies found that the risk differed according to gender [6] or the sites of the fracture [7, 8]. Two meta-analyses related abdominal obesity with a higher risk of hip fracture [9, 10].

There are several mechanisms that can explain this greater bone fragility among obese people: the increased risk of falls [11], the increased impact force in falls due to greater weight, and the deleterious effect of chronic inflammation present in obesity [12, 13]. In addition, in this pathogenic scheme, there is an element that we must take into account: vitamin D. Obese patients have been shown to have lower levels of vitamin D than the lower BMI population [14, 15]. However, some researchers indicate that these low levels of vitamin D are not enough to affect bone health in obese people, for different mechanisms [13, 16]. Nevertheless, no studies in the literature relate obesity, risk of fractures and vitamin D in postmenopausal women. In any case, the link between obesity and osteoporotic fracture is not yet clear and authors agree on the need for more studies to address this issue.

On the other hand, like obese patients, patients with T2DM also have higher BMD values than the population without diabetes, and, despite this, a higher risk of fracture has been reported [17, 18]. Also patients with T2DM have lower levels of vitamin D than the non-diabetic population [19]. However, it is not clear whether these findings can be explained or not by the higher proportion of body fat that patients with T2DM have.

In this study, we considered the effect of obesity on BMD, vitamin D and PTH levels and the risk of fracture in a group of postmenopausal women. Also we investigated the way in which, in obese women, the coexistence of T2DM modifies all these parameters.

## Materials and methods

This is a cross-sectional study carried out on 679 postmenopausal women who were attended at the Bone Metabolic Unit of the Hospital University Insular, Gran Canaria, Spain. For all subjects, a questionnaire, previously validated and used in other similar clinical studies, was completed to gather clinical data on osteoporosis. Obesity was defined as a BMI ≥ 30. The diagnosis of T2DM was made according to the criteria of the American Diabetes Association [20].

### Sample collection and laboratory techniques

Serum levels of 25(OH)-vitamin D were measured by immunochemical luminescence, according to the Nichols method (Nichols Institute Diagnostics, San Clemente, California, USA). Serum parathyroid hormone (PTH) concentrations for the intact molecule were determined by immunochemical luminiscence, according to the Nichols Advantage method.

### Bone mineral density (BMD)

BMD was measured by dual X-ray absorptiometry (DXA), in both lumbar spine (L2–L4) and proximal femur, with a Hologic Discovery® densitometer (Hologic Inc. Waltham, USA). All the measurements were made by the same operator, so there was no inter-observer variation. The *T*-score values were calculated from the values published as normal for the Canary Island population [21].

### Fragility fractures

#### Vertebral fractures:

A lateral thoracic-lumbar X-ray was carried out on the subjects. All the X-rays were brought together and studied by two different observers: one was a radiologist, and the second was an expert on bone metabolic diseases. According to the Genant criteria, the existence of vertebral deformity was stated when there was a reduction in the vertebral height higher than 20% [22].

#### Non-vertebral fractures:

The remaining fragility fractures were confirmed by hospital clinical reports, from the emergency services or by the study of radiographs, excluding self-diagnosis of fractures by the patients.

### Ethics

The study was carried out following the norms of the Declaration of Helsinki [23] and was approved by the Ethics Committee of the Insular University Hospital. All patients were informed of the objectives of the work, and their informed consent was requested.

### Statistical analysis

*Univariate analysis.* Categorical variables are expressed as frequencies and percentages and continuous as mean and standard deviation (SD) when data followed a normal distribution, or as median and interquartile range (IQR = 25th–75th percentile) when distribution departed from normality. The percentages were compared, as appropriate, using the Chi-square (χ^2^) test or the exact Fisher test, the means by the *t* test, and the medians by the Wilcoxon test for independent data.

*Adjusted means by age*. For the DXA markers, adjusted means by age and body mass index (BMI) were obtained using the multivariate least squares regression. Results were summarized as adjusted means (95%CI).

*Adjusted probabilities by age*. By the binary variables (total fragility fractures, vertebral fractures, non-vertebral fractures, hip fractures and falls), the probabilities of each event according presence or not of obesity or T2DM, adjusted by age were estimated using the linear logistic regression.

*Multivariate logistic analysis for the fragility fractures*. In order to identify those factors with independent association with the fragility fractures, a multivariate logistic regression analysis was carried out. The variables that showed significant association the outcome in univariate analysis were entered into the multivariate analysis. Selection of variables based on the best subset regression and Akaike Information Criterion (AIC) was then carried out. The model was summarized as p-values (likelihood ratio test) and odds-ratio, which were estimated by means of confidence intervals at 95%.

Statistical significance was set at *p* < 0.05. Data were analyzed using the *R* package, version 3.6.1 (*R* Development Core Team, 2019) [24].

## Results

### Obesity versus non-obesity

The 679 postmenopausal women who were included in the study were classified into 2 groups: obese and non-obese.

#### Anthropomorphic characteristics, T2DM, falls and fragility fracture

Obese women were older, and their height, weight, and BMI were higher than non-obese women. Furthermore, they also showed a higher prevalence of T2DM, as well as fragility fractures and falls in the last year. When observing the type of fractures, obese women showed a higher percentage of vertebral, hip and non-vertebral fractures, and although the difference was only significant for the latter, for the other types of fracture, vertebral and hip, the level of significance was on the edge. Given that there were significant differences in age, and both falls and fractures were more frequent in older people, the probabilities of each event according to presence or not of obesity adjusted by age were estimated using the linear logistic regression, obtaining as a result that the differences the probabilities ceased to be significant (Table 1).

**Table 1:** Characteristics of the women included in the study: overall and according to BMI

	Overall *N* = 679	*Obesity*		*P*-value
		No *N* = 493	Yes *N* = 186	
Age (years)	60.6 ± 13.6	59.0 ± 13.8	64.8 ± 12.1	< 0.001
Weight (kg)	66.0 ± 13.2	60.4 ± 8.9	81.0 ± 10.9	< 0.001
Height (cm)	156.5 ± 7.2	157.2 ± 7.3	154.6 ± 6.7	< 0.001
Body mass index (kg/m^2^)	27.0 ± 5.3	24.5 ± 3.2	33.8 ± 3.7	< 0.001
Type 2 diabetes mellitus	81 (11.9)	49 (9.9)	32 (17.2)	0.009
Falls	235 (35.0)	159 (32.7) 0.329 (0.287; 0.374)*	76 (40.9) 0.357 (0.290; 0.431)*	0.048 0.496*
Fragility fractures (All)	248 (36.5)	168 (34.1) 0.341 (0.298; 0.387)*	80 (43.0) 0.365 (0.295; 0.440)*	0.031 0.581*
Vertebral fractures	87 (12.8)	61 (12.4) 0.112 (0.086; 0.145)*	26 (14.0) 0.100 (0.066; 0.150)*	0.577 0.633*
Non-vertebral fractures	189 (27.8)	124 (25.1) 0.249 (0.212; 0.291)*	65 (35.0) 0.302 (0.238; 0.373)*	0.011 0.172*
Hip fractures	35 (5.2)	24 (4.9) 0.039 (0.024; 0.062)*	11 (5.9) 0.036 (0.018; 0.070)*	0.583 0.842*
PTH (pg/mL)	45.0 (34.5–60.9)	42.1 (33.0–56.4)	54.9 (39.7–79.5)	< 0.001
25(OH)-vitamin D (ng/mL)	22.8 (16.0–30.8)	23.0 (16.3–31.9)	20.8 (14.4–28.0)	< 0.001

Data are frequencies (%) and medians (IQR)

*Data are adjusted probabilities adjusted by age [95%CI] means (95%CI) by age

#### 25(OH)-vitamin D and PTH

Obese women had lower vitamin D values than non-obese women and higher PTH values, both highly significant differences (Table 1).

#### Bone mineral density

Table 2 shows the results of BMD. The data were adjusted for age. Obese women showed higher BMD values than non-obese women in all the sites measured: lumbar spine L2–L4 and proximal femur (femoral neck and total hip), with a significance level of *p* < 0.001 in all of them. The mean BMD values were all in the ranges of normality or osteopenia.

**Table 2:** Bone mineral density adjusted by age in obese and non-obese women

	Non-obese women	Obese women	*P*-value
Lumbar spine			< 0.001
g/cm^2^	0.831 [0.816; 0.845]	0.905 [0.881; 0.930]	
*T*-score	− 2.014 [− 2.156; − 1.873]	− 1.294 [− 1.527; − 1.062]	
Femoral neck			< 0.001
g/cm^2^	0.653 [0.643; 0.663]	0.736 [0.720; 0.753]	
*T*-score	− 1.715 [− 1.808; − 1.622]	− 0.951 [− 1.103; − 0.799]	
Total Hip			< 0.001
g/cm^2^	0.758 [0.746; 0.770]	0.887 [0.867; 0.907]	
*T*-score	− 1.660 [− c1.787; − 1.533]	− 0.331 [− 0.540; − 0.122]	

Data are adjusted means by age [95% CI] (least squares regression)

### Obesity and T2DM

Once the results were obtained by comparing obese patients with non-obese women, the influence of comorbidity obesity-T2DM was evaluated; for this, obese women were grouped into obese women with T2DM and obese women without T2DM.

#### Anthropomorphic characteristics, falls and fragility fracture

Obese women with T2DM were older and had slightly higher BMI than those without T2DM; they suffered more falls and presented more fragility fractures, although when considering location there were only significant differences in non-vertebral fractures. The probabilities of each event according to presence or not of T2DM adjusted by age were estimated using the linear logistic regression, and as a result the significant differences in fractures were maintained, but not in falls (Table 3).

**Table 3:** Characteristics of the obese women according presence/absence of T2DM

	Obesity *N* = 186	Obese-non-diabetic women *N* = 154	Obese-diabetic women *N* = 32	*P*-value
Age (years)	64.8 ± 12.1	63.9 ± 12.4	68.7 ± 9.7	0.042
Weight (kg)	81.0 ± 10.9	80.6 ± 10.9	82.8 ± 10.4	0.302
Height (cm)	154.6 ± 6.7	154.8 ± 6.9	153.9 ± 5.8	0.491
BMI (Kg/m^2^)	33.8 ± 3.7	33.6 ± 3.5	35.0 ± 4.4	0.046
Falls	76 (40.9)	57 (37.0)	19 (59.4)	0.019
		0.368 (0.293; 0.450)*	0.551 (0.371; 0.718)*	0.069*
Fragility fractures (All)	80 (43.0)	58 (37.7)	22 (68.8)	0.001
		0.369 (0.291; 0.454)*	0.642 (0.450; 0.797)*	0.010*
Vertebral fractures	26 (14.0)	21 (13.6)*	5 (15.6)	0.781
		0.134 (0.088; 0.198)	0.138 (0.057; 0.300)*	0.942*
Non-vertebral fractures	65 (35.0)	45 (29.2)	20 (62.5)	< 0.001
		0.270 (0.200; 0.353)*	0.565 (0.377; 0.736)*	0.004*
Hip fractures	11 (5.9)	9 (5.8)	2 (6.2)	1
		0.039 (0.016; 0.093)*	0.033 (0.007; 0.145)*	0.836*
PTH (pg/mL)	55 (40–80)	56 (43–81)	42 (34–70)	0.184
25(OH)-vitamin D (ng/mL)	20.8 (14.4–28.0)	20.9 (14.3–27.1)	18.4 (14.7–29.0)	0.800

Data are frequencies (%) and medians (IQR)

*Data are adjusted probabilities adjusted by age [95%CI] means (95%CI) by age

#### 25(OH)-vitamin D and PTH

No significant differences were observed in 25(OH)-vitamin D and PTH levels between obese-diabetic women and obese-no-diabetic women (Table 3).

#### Bone mineral density

Table 4 shows the results obtained when comparing BMD values in both groups, obese with and without T2DM, adjusted for age. No significant differences were observed in any of the measured sites between the two groups. The mean BMD values were all in the ranges of normality or osteopenia.

**Table 4:** Bone mineral density adjusted by age in obese-diabetic women and obese-non-diabetic women

	Obese-non-diabetic women *N* = 154	Obese-diabetic women *N* = 32	*P*-value
Lumbar spine			0.197
g/cm^2^	0.903 [0.874; 0.931]	0.858 [0.796; 0.920]	
T-score	− 1.321 [− 1.592; − 1.051]	− 1.751 [− 2.344; − 1.157]	
Femoral neck			0.708
g/cm^2^	0.717 [0.699; 0.736]	0.726 [0.685; 0.767]	
T-score	− 1.127 [− 1.298; −0.956]	− 1.048 [− 1.425; − 0.672]	
Total hip			0.290
g/cm^2^	0.868 [0.846; 0.889]	0.896 [0.848; 0.943]	
T-score	− 0.530 [− 0.750; − 0.310]	− 0.239 [− 0.730; 0.252]	

Data are adjusted means by age [95% CI] (*least squares regression*)

### Multivariate logistic regression for fragility fractures

It was observed that, of all the data entered in the analysis, the logistic regression model for the prediction of fragility fractures in the total group only selected 4 independent parameters: age, BMI, T2DM and BMD in total hip. The most powerful association from the statistical point of view was that obtained with T2DM. The other associations, although statistically significant, showed lower odd ratio (OR) values: BMI, age, and total hip BMD, the latter being the only factor with an inverse effect (less BMD, more risk of fracture). However, when applying the Akaike Information Criterion (AIC), it was obtained that total hip BMD is the variable that has the greatest influence on the model, followed by age, BMI and T2DM (Table 5).

**Table 5:** Multivariate logistic regression for fragility fractures

	Coefficient (SE)	*P*-value *	AIC**	Odd-Ratio (95% CI)
Age, per year	0.034 (0.008)	< 0.001	691.0	1.035 (1.018; 1.052)
Body mass index, per kg/m^2^	0.085 (0.021)	< 0.001	689.3	1.089 (1.045; 1.135)
Type 2 diabetes mellitus	0.786 (0.286)	0.006	680.4	2.196 (1.253; 3.846)
Total hip, per g/cm^2^	−4.771 (0.818)	< 0.001	711.0	0.008 (0.002; 0.042)

when the fit improves, the AIC decreases

If the age of the model were omitted, the greatest worsening of the fit would occur; this means that “*Total hip*” is the variable with the greatest influence

*Likelihood ratio test

**If the factor is removed; AIC for the full model = **674.8**; AIC is a measure of lack of fit of the model to the data;

## Discussion

In our study, as expected and according to what was obtained by most other studies, obese women showed higher BMD values than non-obese women, but this higher BMD did not have a protective effect on the risk of fracture, which might indicate that obesity somehow counteracts that effect.

A meta-analysis by Kaza et al. concluded that BMI was associated with an increased risk of vertebral fracture in women (after adjustment for BMD), but it was decreased in men [6]. Various studies point to obesity as a risk factor for fractures in certain locations, despite higher levels of BMD. In a meta-analysis carried out by Johansson et al. [7], the authors concluded that obesity by itself is not a risk factor for osteoporotic fracture, including those of the hip or forearm, and that there is an inverse association between BMI and risk of fracture, dependent on the BMD. However, they suggested that the association is specific to the fracture site and that obesity in postmenopausal women protects against osteoporotic fractures, but is associated (albeit weakly) with an increased risk of tibia/fibula fractures and of humerus/elbow. In their analysis, the authors do not assess falls, although they do consider them as an explanation for this association.

Compston et al. [5] studied the incidence and prevalence of fractures in a large number of postmenopausal women, reporting a lower risk of wrist fracture, but a higher risk of incidence of ankle fracture and upper leg fracture among obese women (also weak association), also pointing to their higher prevalence of falls as a factor etiopathogenic.

Court-Brown et al. [25] evaluated the BMI in a group of 4886 adult patients of both sexes who had suffered a fracture, finding a negative or positive association between obesity and fractures depending their locations and individuals gender. Similar results were reported by Lacombe et al. [8] and Prieto-Alhambra et al. [26] studying postmenopausal women. In a recent study, Adami et al. [27] used a new web-based fracture risk-assessment tool that collects demographic and clinical data from a large number of women (59,959), analyzing the risk of fractures (grouped in prevalent vertebral, hip, and non-vertebral non-hip fractures) in obesity and diabetes. Regarding obesity, the analysis showed that obese women had more BMD in femoral neck and lumbar spine compared to non-obese women and that there was a weak association only between non-vertebral non-hip fractures with obesity.

The results of our study regarding the association between fractures and obesity are in line with those of the referred studies, weak positive association in certain locations that are not typically osteoporotic (vertebral, hip and forearm fractures). Although some authors have focused on falls as the possible explanation, in our study we did not find a higher probability of falls in obese women, whose higher prevalence was explained by age.

We have not found any studies linking obesity, 25(OH)-vitamin D levels and fractures, as has been done in this study. Obese women studied had lower levels of 25(OH)-vitamin D than non-obese women and significantly, as expected coinciding with other studies [28, 29]. However, it must be borne in mind that the average level of vitamin D in the total group of women studied was low, 22.8 ng/mL, so both obese and non-obese were close to vitamin D insufficiency, if we look at the generally accepted limit of 20 ng/mL. In this way, the negative effect of hypovitaminosis D would be present in both groups, without it being able to produce clear influences on the risk of fracture. The finding of higher levels of PTH in response to the lower values of 25(OH)-vitamin D in obese women found in this study also coincides with that reported by other authors [28, 29]. The mean levels of PTH in the women in our study were, in addition, higher than the normal values for our test (*N* = 9–40 pg/mL), which is indicative of an hyperparathyroidism secondary to insufficient vitamin D to maintain calcium levels, which was shown to be within normality, as other authors have observed [29]. A study by Sukumar et al. [30] in women aged 25–71 years reported that obese women had lower values of 25(OH)-vitamin D and higher values of PTH and that the volumetric trabecular BMD was increased, but due to the action of hyperparathyroidism the volumetric cortical BMD was decreased, but without compromising bone geometry and strength. The authors do not analyze the impact on the risk of fracture.

In this study, we wanted to know whether T2DM induced alterations in the parameters studied in obese women. Although obese women with T2DM had more falls per year than non-diabetics, as was the case when comparing obese with non-obese, this difference was attributed to age. However, the prevalence and probability of fractures after adjusting for age was significantly higher, specifically for non-vertebral fractures.

This finding was interesting to us. Somehow, T2DM poses an added risk in obese women to suffer fractures, specifically non-vertebral ones. We did not find an explanation for these results from the parameters studied, but it is possible that some or more of the pathways indicated in the introduction could be responsible for this greater bone fragility. On the other hand, in our study we did not find significant differences in BMD of any of the sites measured between obese-diabetic and obese-non-diabetic women, possibly because the higher BMD of diabetic patients is due to associated obesity.

In the aforementioned study by Adami et al. [27], diabetic women had higher BMD values than non-diabetic women (without taking BMI into account), and diabetes, when excluding obese women and adjusting for age, was associated with all types of fracture, vertebral or hip fractures and non-vertebral non-hip fractures. When they analyzed the diabetes-metabolic syndrome comorbidity, they obtained as a result that diabetic-obese women had higher BMD values in lumbar spine (not in femoral neck), than obese-non-diabetic women. Prevalence of fractures was higher in obese-diabetic women and obese-non-diabetic women in all locations, and only in non-vertebral/non-hip fractures in diabetics-non-obese, compared to normal weight and non-diabetic women. They did not compare obese-diabetic women with obese-non-diabetic women, as we did in our work.

The levels of 25(OH)-vitamin D and similar PTH in diabetic-obese and non-diabetic-obese women lead us to conclude that in our study the hypovitaminosis D and secondary hyperparathyroidism that our patients with T2DM present are determined for associated obesity.

The multivariate logistic regression analysis agrees and is consistent with the results obtained and further defines the role of obesity in the risk of fracture. It is well recognized that BMD and age are the most important predictors of fracture risk, as we have obtained in our model, but we are strongly struck by the fact that in our model BMI and T2DM are considered independent factors of fracture risk (and both increasing the risk), although T2DM shows more predictive power than BMI. It is important to note that the women in our study generally did not have a lower than normal BMI, which is considered a risk factor for fracture, so it is not considered in the predictive model. As we have said before, this predictive model is not entirely contrary to the results obtained in the comparison of the groups, but rather, in some way, helps to understand and explain it: while the total hip BMD, the variable more influential, has a reverse and protective effect (more BMD lower risk), older age, higher BMI and the presence of T2DM increase the risk. Our obese women have more total hip BMD, but also are older and have higher BMI and greater presence of T2DM, and the opposite effects of these variables may make the risk of fracture not affected either positively or negatively.

Our study has strengths and weaknesses: among the former, the large number of variables studied, which has allowed us to show a broader vision of the issue; on the other hand, the number of patients studied gives consistency to most of the results; as a weakness, the sample size of the fractures produced in the population studied, which may have influenced that the results in this regard are not more significant.

Our results show consistency to be able to affirm that obese postmenopausal women have higher BMD values and lower than 25(OH)-vitamin D (associated with higher PTH levels) than non-obese women, without it seems that diabetes influences these parameters. Regarding the production of fractures, our results do not confer on obesity a protective role of the risk of fracture, but rather it favors them, although its influence is not powerful, nor dependent on BMD or vitamin D. However, diabetes clearly does confer an increased risk of fractures, at least non-vertebral ones. Hypovitaminosis D does not seem to influence risk fractures.

In conclusion, in obese postmenopausal women, we did not observe an increased risk of fragility fracture, but the association of type 2 diabetes mellitus significantly increased the risk of these fractures, specifically non-vertebral fractures.

## Summary box

### What is known

There is an association between obesity and type 2 diabetes mellitus. Obese postmenopausal women reportedly present higher values of bone mineral density. Even so, it has been described that obesity and T2DM increase the risk of suffering fragility fractures, although other studies do not report this increased risk.

### What is the question

Are obese postmenopausal women at higher or lower risk of fragility fractures? Does the comorbidity of obesity and T2DM affect this risk?

### What was found

Obese postmenopausal women had higher values of BMD than non-obese women, but there was no difference in fracture risk. Diabetic-obese women had a higher prevalence of fragility fractures than non-diabetic-obese. Fragility fractures were associated to age, BMD, BMI and T2DM.

### What is the implication for practice now

We should not confer on obesity a protective role of fracture risk. Indeed, comorbidity of obesity and T2DM increase the risk of suffering from non-vertebral fragility fractures.
